# Long-term results of total knee arthroplasty with single-radius versus multi-radius posterior-stabilized prostheses

**DOI:** 10.1186/s13018-019-1183-0

**Published:** 2019-05-16

**Authors:** Zhenyu Luo, Zeyu Luo, Haoyang Wang, Qiang Xiao, Fuxing Pei, Zongke Zhou

**Affiliations:** Department of Orthopedics, West China Hospital/West China School of Medicine, Sichuan University, 37# Wuhou Guoxue road, Chengdu, 610041 People’s Republic of China

**Keywords:** Total knee arthroplasty, Single-radius, Multi-radius, Long-term results, Anterior knee pain

## Abstract

**Background:**

Single-radius (SR) prostheses and multi-radius (MR) prostheses have different theoretical advantages; however, there has been a paucity of evaluations comparing the two. This study was designed to compare the 10-year clinical, radiological, and survival outcomes of SR and MR posterior-stabilized prostheses in total knee arthroplasty (TKA).

**Methods:**

In this retrospective cohort study, 220 consecutive patients undergoing TKA between October 2006 and October 2007 were divided into the SR group (106 patients, Stryker Scorpio NRG) and the MR group (114 patients, DePuy Sigma PFC), with a minimum follow-up of 10 years. Clinical, functional, and radiological outcomes, as well as satisfaction rates and survival results, were evaluated.

**Results:**

Hospital for Special Surgery and Short Form-12 health survey scores were all significantly improved in both groups at the final follow-up (*P* < 0.05), but the groups did not differ. The SR group had significantly less anterior knee pain (AKP) and painless crepitation (*P* < 0.05). Radiological results in terms of radiolucent lines and component position angle showed no differences between groups. The Kaplan-Meier survival curve estimates at 10 years were not significantly different between the groups (*P* = 0.4172).

**Conclusion:**

Both SR and MR posterior-stabilized prostheses can lead to satisfactory outcomes. The SR prosthesis design gave less anterior knee pain than did the MR prostheses. Two prostheses showed no differences in terms of clinical scales, radiological results, satisfaction rates, and survival results at a long-term follow-up. More accurate measurements are required.

## Background

Total knee arthroplasty (TKA) excellent operation rate has reached 90% [1], and the revision rate has been less than 4% [2]. However, the satisfaction rate of patients with TKA has only been 75–80%, and 10% of patients still experience anterior knee pain (AKP) or patellofemoral complications [3].

The first theory of the knee rotation center was proposed by Frankel et al. [4] in 1971. They described knee flexion occurring around a varying transverse axis, with the instantaneous rotation center of the femoral posterior condyle forming a “J curve” [4, 5]. The multi-radius (MR) femoral prosthesis was theoretically designed, and the trans-epicondylar axis (TEA) which was perpendicular to the mechanical alignment (MA) of the lower extremity was widely used as a skeletal marker for the axis of flexion and rotation [6]. The multi-radius femoral prosthesis was designed based on this theory and was widely utilized since the 1980s. For example, in the sigma PFC system, the femoral implant had three segment progressive radius at the sagittal plane, and in the recent MR prostheses, the Attune system has been a continuously progressive radius like a spiral cord which may provide a more fluent flexion movement [7].

However, in 1993, Hollister et al. [8] demonstrated that the actual flexion-extension axis (FEA) of the knee sometimes did not coincide with the epicondylar axis and had a more distal and posterior axis. The locus of the rotation center between 10 and 120° can be regarded as a single spherical radius [8, 9]. The single-radius (SR) femoral prosthesis was designed according to this theory. For example, the first SR prosthesis, the Scorpio single-radius TKA (Stryker, Mahwah, NJ), was produced in 1996 [10]. The second stage was the Scorpio NRG system, and it is now the latest stage with high-flexion system in the Triathlon TKA prosthesis [11, 12].

The SR prosthesis provided a more distal FEA to have a longer extensor moment arm [13], maintaining stabilization during the middle segment range of motion (ROM), thereby reducing the paradoxical anterior femoral movement [14], alleviating anterior knee pain (AKP), and providing a better patellofemoral trajectory [11]. Although SR prosthesis designs had theoretical advantages, there has been lack of sufficient quality studies. Most of these studies included no more than 50 patients in each group or followed up for no longer than 3 years [15–18], as shown in Table 5. Finally, some studies suffered from methodological shortcomings.

The aim of this retrospective cohort study was to compare the clinical results between SR and MR posterior-stabilized (PS) prostheses. We hypothesized that both two prostheses can provide satisfactory results and the SR prostheses provide similar functional, radiological, and survival results to the MR prostheses in TKA.

## Methods

The retrospective cohort study was approved by the Institutional Review Board of West China Hospital, Sichuan University (ID number: 2012-268). The work was registered in the Chinese Clinical Trial Registry (ID number: ChiCTR1800016129). Informed consent was obtained from all patients and their relatives. Information about deceased participants was collected from their relatives.

This retrospective cohort study included a consecutive series of 327 patients who underwent TKA by one surgery group in West China Hospital between October 2006 and October 2007. We included patients who underwent primary unilateral TKA with SR (Stryker Scorpio NRG) or MR (DePuy sigma PFC) prostheses. The two prostheses were the most utilized prostheses during that time period. We declared no additional information about the implants to patients, so which implant to utilize was irrelevant to characteristics of the patients or severity of the symptoms and was only chosen by patients themselves for their own desires.

**Fig. 1:**
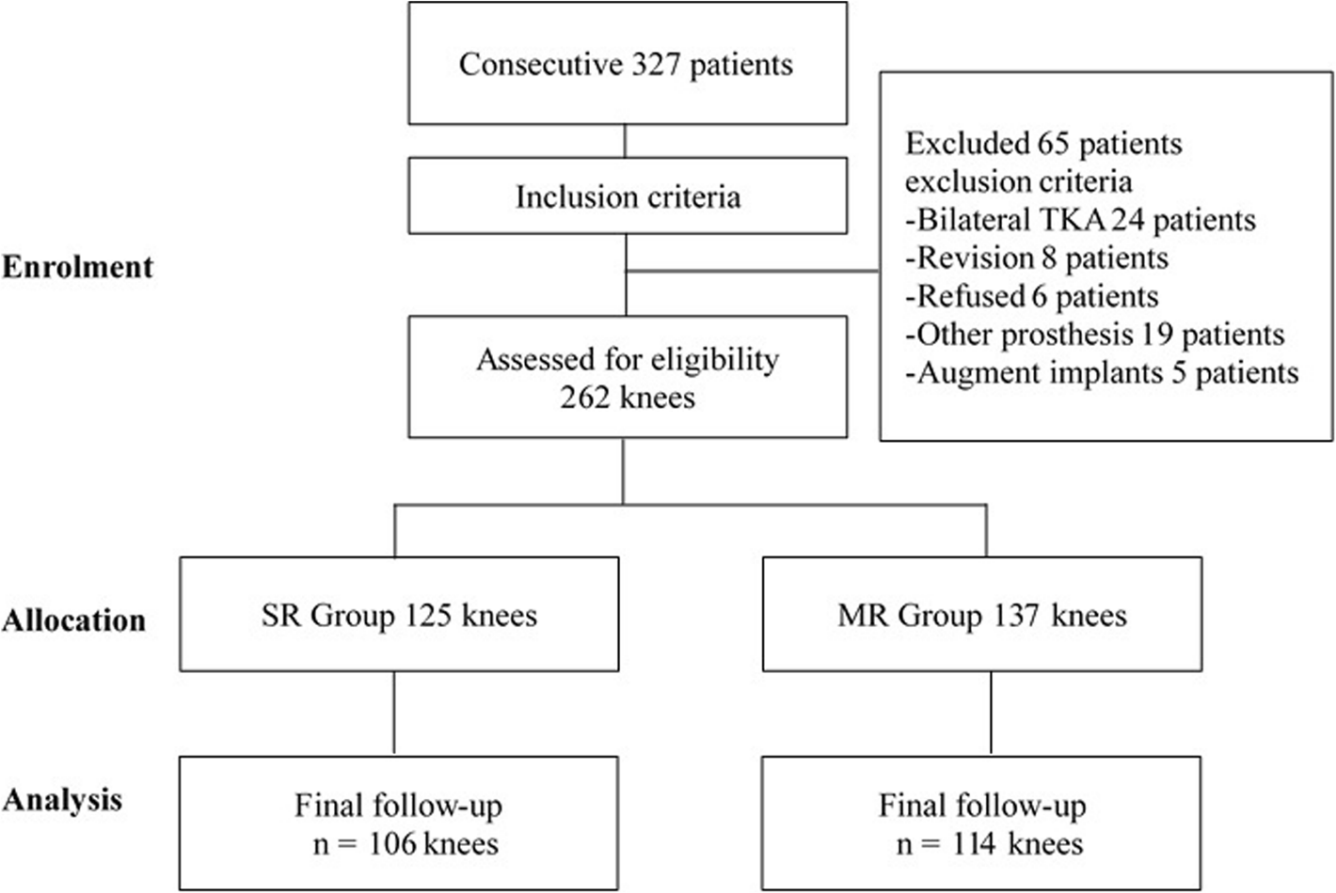
Flow diagram for trial participation

We excluded patients with revision TKA, patients diagnosed with inflammatory arthritis, patients who underwent bilateral TKA, patients who required cruciate-retain (CR) or any other types of SR or MR prosthesis, patients who required cones or augments for severe bone defects, and patients who could not give informed consent. Therefore, 65 patients were excluded from the study for any reasons (Fig. 1). At the final 10-year follow-up, 42 patients were lost to follow-up, underwent revision, or died (Table 3). Accordingly, the final follow-up was conducted on 220 patients, among whom 106 received SR prostheses and 114 patients received MR prostheses. Baseline data from the two groups are listed in Table 1. There were no significant differences in terms of mean age, gender, BMI, or diagnoses of disease.

**Table 1:** Demographic data of patients at final follow-up

	SR group	MR group	*P* value
Patients	106	114	–
Gender (female/male)	82/24	78/36	0.1369
Age	69.54 ± 10.57	68.98 ± 10.23	0.8418
BMI	23.38 ± 2.58	23.45 ± 2.61	0.6901
Follow-up years	10.72 ± 1.78	10.65 ± 1.87	–
Diagnoses			
Primary osteoarthritis	81 (76.74%)	85 (78.31%)	0.7496
Rheumatic arthritis	20 (17.44%)	23 (15.66%)	0.8069
Posttraumatic arthritis	2 (2.33%)	2 (2.41%)	0.3769
Gouty arthritis	3 (3.49%)	4 (3.61%)	0.2864

Continuous data presented as the mean ± std. Student’s *t* test was utilized. Discontinuous data presented as frequencies (percentages). Pearson’s chi-squared test or Fisher’s exact test was utilized. *P* < 0.05 indicates significant differences

### Surgical technique

One surgery team performed all operations. The SR prostheses were Scorpio NRG (Stryker Orthopedics, Mahwah, NJ, USA) and the SR prostheses were Sigma PFC (DePuy Orthopedics, IN, USA). All K-L (Kellgren-Lawrance) arthritis grades of the patients were in grade IV. A medial parapatellar approach was used. Distal femoral osteotomies were usually 6° to the femur anatomic axis [19]. Femoral rotational alignment was performed according to the epicondylar axis, usually 3° of external rotation from the posterior condylar line. Both groups removed the posterior cruciate ligaments (PCL). Femoral components were similar size (standard size) in both designs, and the cemented tibial baseplates were also similar in both designs. Patellar resurfacing was not utilized in either group. All components were cemented. All patients were given cephalosporin for 24 h to prevent infection. A plasma drainage tube was used for 24 h. Continuous movement exercises were started postoperatively to recover quadriceps strength.

### Evaluation

Patients were regularly followed up every 6 months after surgery. The evaluation was conducted using HSS (Hospital for Special Surgery) scores, KSS (Knee Society score, knee and function), WOMAC (The Western Ontario and McMaster Universities) score, SF-12 scale (the Short Form-12 health survey, physical and mental), and ROM (range of motion, flexion and extension). The VAS (visual analog scale) and incidence of anterior knee pain and crepitation were recorded. The chair test was used to exam the stability of the prosthesis indirectly, allowing patients to stand from a chair without the assistance of both arms on the armrests [10]. The satisfaction rate was also evaluated.

**Fig. 2:**
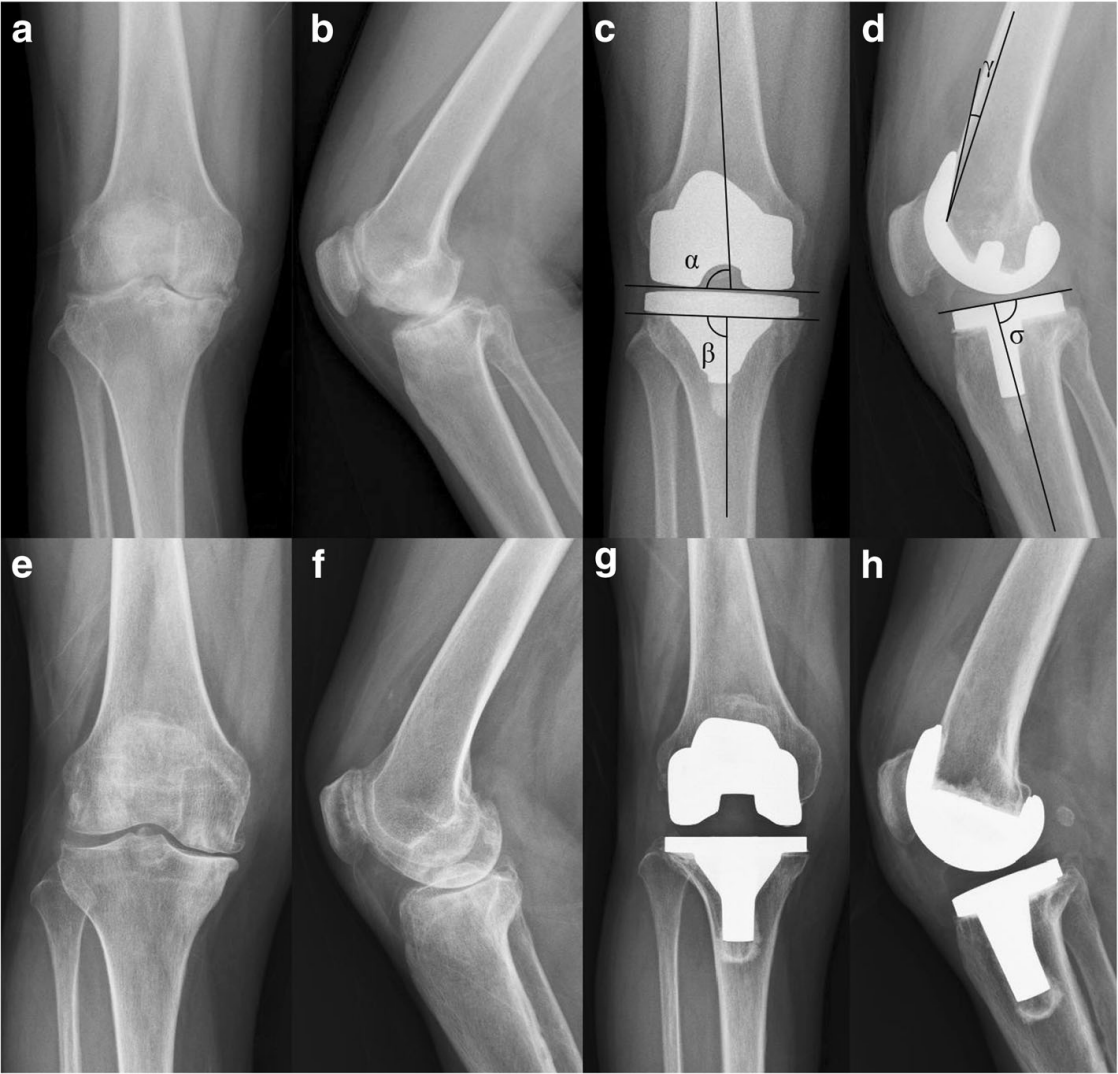
**a**–**d** A 68-year-old female diagnosed with osteoarthritis underwent left knee arthroplasty with an SR (Scorpio NRG) prosthesis. **a**, **b** Preoperatively. **c**, **d** At 10-year follow-up. **e**–**h** A 65-year-old female diagnosed with osteoarthritis underwent right knee arthroplasty with an MR (PFC) prosthesis. **e**, **f** Preoperatively. **g**, **h** At 10-year follow-up. All components were considered stable, and no radiolucent lines or osteolysis were detected. **c**, **d** Radiological evaluation angles

Radiological evaluation was based on weight-bearing standing posterior-anterior, lateral view radiographs. Location of radiolucent lines was evaluated based on the Knee Society Roentgenographic Evaluation System [20]. The component positions were evaluated by four angles: the lateral distal femoral angle (LDFA, α angle), which is measured between the anatomical axis of the femur and a tangential line to the distal condyles of the femoral prosthesis; the medial proximal tibia angle (MDTA, β angle), which lies between the anatomical axis of the tibia and a tangential line to the plateau of the tibial prosthesis; the flexion-extension femoral angle (FEFA, γ angle), which is between a line through the midshaft of the femur and the neutral line of the femoral prosthesis; and the tibial slope angle (TSA, σ angle), which is described by a line through the midshaft of the tibia and a tangential line to the tibial prosthesis [21] (Fig. 2c, d).

Kaplan-Meier survival curves were used to analyze survivorship and used the following end points: (1) death and (2) prosthesis revision for infection, radiographic loosening, or any other reasons with any components.

### Statistical analysis

All continuous data were presented as mean ± standard deviation (ranges). Two-sided, paired Student *t* tests were utilized to analyze preoperative and last follow-up data in both groups. Two-sided, independent sample Student *t* tests were utilized to analyze data between the two groups. All discontinuous data were presented as frequencies (percentages) and were analyzed by Pearson *χ*^2^ tests or Fisher’s exact probability tests. The Wilcoxon rank-sum test was used to analyze ranked data. The Kaplan-Meier survival curves were compared with the Mantel-Cox log-rank test. All significance levels were set at *α* = 0.05, and *P* < 0.05 indicated significant difference. All statistical analysis was calculated using SAS 9.4 (Statistical Analysis System, SAS Institute, Cary, NC, USA).

## Results

### Clinical and functional results

The duration of follow-up was 10.72 ± 1.78 years (range 10–12) in the SR group and was 10.65 ± 1.87 years (range 10–12) in the MR group. There were no significant differences in mean age, gender, BMI, or diagnoses of disease, as shown in Table 1.

All functional results of the two groups are listed in Table 2. No preoperative results differed between the two groups. Both groups showed significant improvements from preoperative values to their follow-up in terms of HSS, KSS, WOMAC score, SF-12, and ROM (flexion and extension). No significant differences were found between the groups. At the final follow-up, no significant differences were found in chair test results between the groups (*P* = 0.7799), as shown in Table 3.

**Table 2:** Summary of patients lost

Reason for drop out	SR group	MR group	Total
Withdrew (not contactable)	12	14	26
Revised	4	5	9
Death	3	4	7
Total	19	23	42

**Table 3:** Results at final follow-up

	SR (*n* = 106)	MR (*n* = 114)	*P* value
Functional results			
HSS			
Preoperative	41.23 ± 5.76	42.35 ± 5.34	0.1359
Final follow-up	86.32 ± 10.22	84.52 ± 10.53	0.2002
KSS (knee)			
Preoperative	40.56 ± 15.62	42.63 ± 17.36	0.5700
Final follow-up	84.52 ± 18.50	85.63 ± 16.82	0.6416
KSS (function)			
Preoperative	45.62 ± 16.57	44.58 ± 17.56	0.6517
Final follow-up	86.23 ± 17.50	85.14 ± 16.20	0.6319
WOMAC score			
Preoperative	54.68 ± 18.69	56.23 ± 17.52	0.5261
Final follow-up	21.83 ± 15.60	23.24 ± 15.80	0.5064
SF-12			
Preoperative	28.84 ± 6.45	30.13 ± 6.14	0.1300
Final follow-up	52.48 ± 5.34	51.21 ± 5.35	0.1394
ROM (flexion)			
Preoperative	105.52 ± 10.78°	104.18 ± 10.62°	0.3542
Final follow-up	115.65 ± 10.32°	115.50 ± 10.13°	0.9135
ROM (extension)			
Preoperative	10.23 ± 2.45°	10.34 ± 2.23°	0.7277
Final follow-up	3.42 ± 1.22°	3.28 ± 1.25°	0.4020
Chair test (complete)	88 (83.02%)	93 (81.58%)	0.7799
Pain			
VAS			
Preoperative	6.45 ± 1.25	6.57 ± 1.38	0.5009
Final follow-up	0.42 ± 0.15	0.44 ± 0.12	0.2744
Anterior knee pain	9 (10.38%)	20 (17.54%)	0.0251*
Painless crepitation	17 (16.34%)	26 (28.07%)	0.0383*
Painful crepitation	2 (1.89%)	3 (2.63%)	0.3287
Component evaluation			
Radiological line			
< 2 mm	8 (7.69%)	10 (8.77%)	0.1842
> 2 mm	0 (0%)	0 (0%)	–
LDFA (α) °	84.32 ± 3.15	84.57 ± 3.25	0.5635
MDTA (β) °	89.87 ± 3.24	89.56 ± 3.32	0.4846
FEFA (γ) °	6.54 ± 1.68	6.76 ± 2.26	0.4162
TSA(σ) °	88.25 ± 3.53	87.82 ± 3.72	0.3809

*Continues data presented as the mean ± std, Student’s *t* test was utilized. Discontinuous data presented as frequencies (percentages); Pearson’s chi-squared test or Fisher’s exact test was utilized. *P*  <  0.05 indicates significant differences

### Pain and crepitation

The SR and MR groups showed no significant differences in VAS scores preoperatively (*P* = 0.5009). At the final follow-up, VAS scores decreased significantly in the two groups. There were no significant differences between the two groups. Nine (10.38%) patients in SR reported AKP, and 17 (16.34%) reported painless crepitation, significantly less than those in the MR group, with 20 (17.54%) patients reporting AKP and 26 (28.07%) reporting crepitation (*P* < 0.05). Severe isolated AKP had no significant differences at the final follow-up.

### Radiological results

At the final 10-year follow-up, in 220 patients, radiolucent lines were found in 8 patients (7.69%) in the SR group and in 10 patients (8.77%) in the MR group, not significantly different (*P* = 0.1842), and all patients in both groups who had radiolucent lines were less than 2 mm. No aseptic loosening of the tibial or femoral component, osteolysis, or infection were observed in either group (Fig. 2). At the final follow-up, angles measuring the component position showed no significant differences between the groups (Table 3).

### Satisfaction rate

At the final follow-up, 76 (71.70%) patients in the SR group were very satisfied and 23 (21.69%) patients were satisfied. In the MR group, 74 (64.91%) patients were very satisfied and 31 (27.19%) patients were satisfied. Rank data in cross-tabulation (Table 4) with Wilcoxon rank-sum test showed no significant differences between the two groups (*Z* = 0.2091, *P* = 0.4172).

**Table 4:** Satisfaction rate at final follow-up

	SR (*n* = 106)	MR (*n* = 114)	Total
Very satisfied	76	74	150
Satisfied	23	31	54
Uncertain	5	7	12
Unsatisfied	2	2	4
Total	106	114	220

Rank data cross tabulation table; discontinuous rank data presented as frequencies; according to Wilcoxon rank-sum test, *Z* = 0.2091, *P* = 0.4172, which indicates no significant differences

### Complication and survivorship

**Fig. 3:**
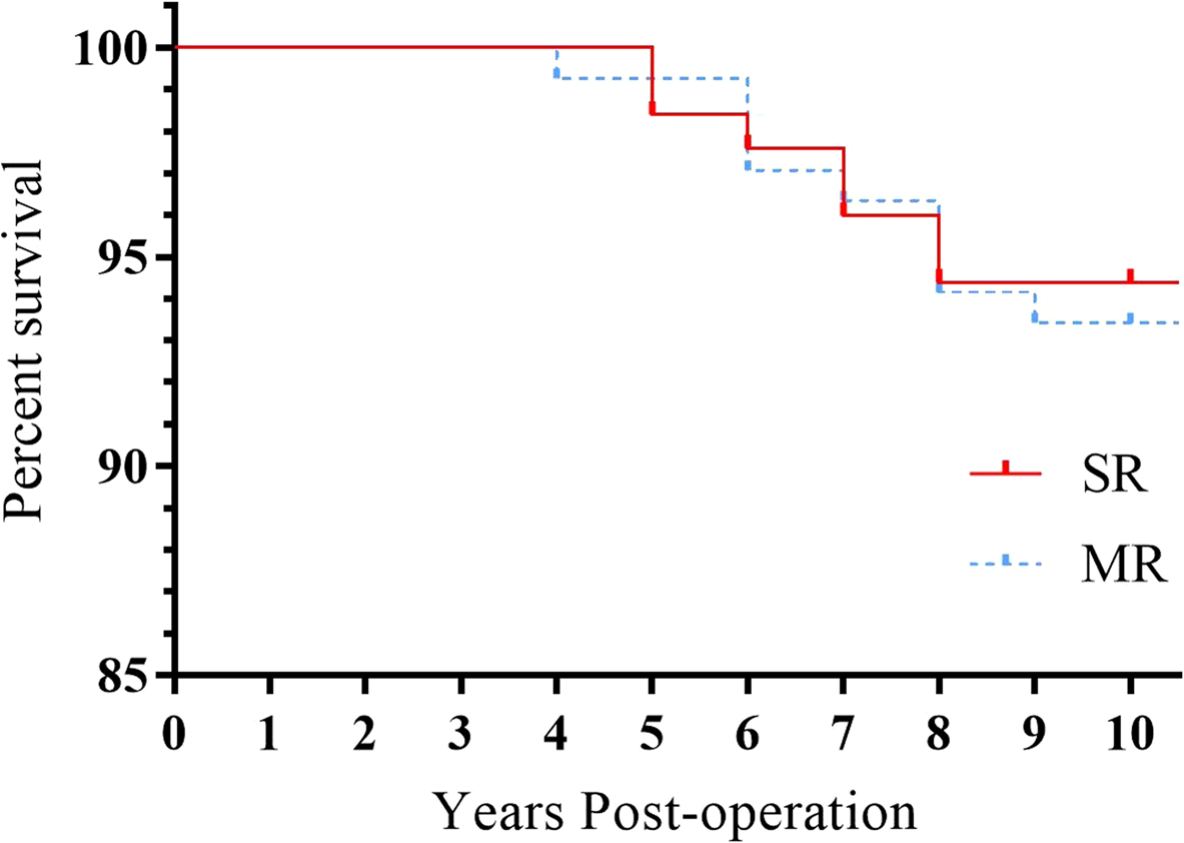
Kaplan-Meier survival curve

During the follow-up period, 12 patients withdrew in the SR group; 3 patients died at 5, 6, and 8 years, respectively; 1 patient underwent revision for periprosthetic fracture at 5 years; 2 patients underwent revision for prosthesis loosening at 7 years; and 1 patent underwent revision for prosthesis loosening at 8 years. Fourteen patients withdrew in the MR group; 4 patients died at 6, 7, 8, and 8 years, respectively; 1 patient underwent revision for periprosthetic fracture at 4 years; 2 patients underwent revision for prosthesis loosening at 6 years; 1 patient underwent revision for prosthesis loosening at 8 years; and 1 patient underwent revision for periprosthetic fracture at 9 years, as shown in Table 2. Kaplan-Meier survival estimated at 10 years was 94.40% (95% CI 90.4–98.4%) in the SR group and was 93.43% (95% CI 89.4–97.4%) in the MR group. There were no significant differences between the two groups by the Mantel-Cox log-rank test (*χ*^2^ = 0.09997, *P* = 0.7519, Fig. 3).

## Discussion

We compared two types of posterior-stabilized prostheses: SR (Scorpio NRG, Stryker Orthopedics, Mahwah, NJ, USA) and MR (PFC, DePuy Orthopedics, IN, USA). We hypothesized that both the two prostheses can provide satisfactory results and the SR prostheses provide similar functional, radiological, and survival results to the MR prostheses. We discovered that the SR prostheses gave less anterior knee pain than did the MR prostheses, while other clinical and functional outcomes did not show any significant differences. Radiological results, complications, and survival of the prostheses were also similar.

We overviewed recent literature of the comparison between SR and MR prostheses and compared our current study to them. As is shown in Table 5, many of the previous studies had conflicting outcomes, none of them provided longer than 5 years follow-up [15–18, 22]. Although our study was a retrospective cohort study, it was the longest study which included at least 10-year follow-up and examined clinical, functional, radiological, and survival outcomes.

**Table 5:** Overview of relevant literature

First author	Year	Design	Origin	Minimal follow-up (M)	Participants		Prostheses	Postoperative results			Complication (required revision)	Survival	Conclusion
					SR	MR		SR	MR	*P* value			
Molt	2012	PRCT	Sweden	24	25	25	SR: Stryker Triathlon, CR MR: Stryker Duracon, CR	KSS knee, 61.84 ± 2.62 KSS function, 86.94 ± 6.33 Mean RSA, 0.76 ± 0.18	KSS knee, 63.84 ± 3.67 KSS function, 88.96 ± 6.33 Mean RSA, 0.63 ± 0.26	> 0.05 > 0.05 0.462	N/A	SR, 100% MR, 100%	SR has similar early stability and same function to MR
Jo	2014	PRCT	South Korea	24	50	50	SR: Stryker Scorpio NRG, CR MR: Zimmer NexGen, CR	HSS, 90.1 ± 6.2 WOMAC, 21.9 ± 8.2 ROM, 128.7 ± 11.6 Stability stress at 30°, 7.6	HSS, 91.9 ± 7.0 WOMAC, 20.2 ± 12.9 ROM, 126.0 ± 12.7 Stability stress at 30°, 8.3	0.42 0.13 0.26 < 0.01	N/A	SR, 100% MR, 100%	SR better intra-operative stability at 30° of flexion. Clinical results no differences in two groups
Palmer	2014	RCS	Australia	24	338	674	SR: Stryker Scorpio NRG, CR MR: Stryker Duracon, CR	KSS knee, 89.5 ± 10.3 KSS function, 71.7 ± 21.5 ROM, 108.5 ± 12.5 No pain, 66.3%	KSS knee, 83.5 ± 14.8 KSS function, 68.7 ± 23.0 ROM, 106.5 ± 13.7 No pain, 54.4%	< 0.05 0.046 0.02 < 0.05	N/A	SR, 100% MR, 100%	SR better knee flexion, knee score and function score than MR
Hamilton	2015	PRCT	UK	36	90	75	SR: Stryker Triathlon, CR MR: Stryker Kinemax, CR	OKS (change), 17.1 (− 14 to 33) ROM, 108.5 ± 9.7 Lower limb power, (94 ± 34)% Worst daily pain, 1.7 ± 1.5 Average pain, 0.5 ± 1.2	OKS (change), 20.1(−3 to 39) ROM, 99.8 ± 9.7 Lower limb power, (116 ± 40)% Worst daily pain, 2.5 ± 2.2 Average pain, 0.9 ± 1.4	0.05 < 0.05 < 0.05 < 0.05 0.57	SR: 1 early infection MR: 2 early infection, 1 revised	N/A	SR better function (lower limb power and knee flexion), pain levels, and overall satisfaction
Oliviu	2016	RCS	Romania	32	94	70	SR: Stryker NRG, PS MR: Zimmer NexGen, PS	KSS knee, 87.2 ± 7.5 KSS function, 86.2 ± 7.1	KSS knee, 86.8 ± 11.3 KSS function, 81.5 ± 13.7	> 0.05 > 0.05	SR: 1 apparent and 1 impending mechanical failure MR: 1 impending mechanical failure	SR, 95.8% (95% CI 91.8–99.8%) MR, 92.7% (95% CI 87.7–97.7%) (at 60 months, *P* = 0.31)	No differences in two groups.
Isabel	2017	PRCT	Spain	60	118	119	SR: Italy Samo, CR MR: Italy Lima, CR	KSS knee, 83.9 ± 6.6 KSS function, 84.2 ± 8.5 ROM, 105.2 ± 10.0 Quadriceps strength, 3.0 ± 1.2 Chair test, 103/15	KSS knee, 83.9 ± 6.6 KSS function, 84.2 ± 8.5 ROM, 99.4 ± 8.1 Quadriceps strength, 2.6 ± 0.9 Chair test, 92/27	0.001 0.001 0.001 0.004 0.032	SR: 1 deep wound infection, 2 aseptic tibial loosening MR: 1 deep wound infection, 2 aseptic tibial loosening; 1 periprosthetic femoral fracture	SR, 97.4% (95% CI 92.4–100%) MR, 97.4% (95% CI 94.6–100%) (at 60 months, *P* > 0.05)	SR shows better results than MR. The use of SR system is recommended
This study	2018	RCS	China	120	106	114	SR: Stryker NRG, PS MR: Sigma PFC, PS	HSS, 86.32 ± 10.22 KSS knee, 84.52 ± 18.50 KSS function, 86.23 ± 17.50 WOMAC, 21.83 ± 15.60 ROM, 115.65 ± 10.32° Anterior knee pain, 9 (10.38%) Chair test, 88 (83.02%)	HSS, 84.52 ± 10.53 KSS knee, 85.63 ± 16.82 KSS function, 85.14 ± 16.20 WOMAC, 23.24 ± 15.80 ROM, 115.50 ± 10.13° Anterior knee pain, 20 (17.54%) Chair test, 93 (81.58%)	0.200 0.570 0.614 0.632 0.501 0.025 0.780	SR: 1 periprosthetic fracture, 3 prosthesis loosening; MR: 2 periprosthetic fracture, 3 prosthesis loosening	SR, 94.4% (95% CI 90.4–98.4%) MR, 93.4% (95% CI 89.4–97.4%) (at 120 months, *P* = 0.7519)	SR less anterior knee pain than MR. Clinical, radiological, and survival results no differences in two groups.

*PRCT* prospective randomized controlled trial, *RCS* retrospective cohort study, *HSS* Hospital for Special Surgery scores, *KSS* Knee Society score, *WOMAC* The Western Ontario and McMaster Universities score, *OKS* Oxford knee score, *ROM* range of motion < 0.05 indicates significant differences

Although TKA had wonderful results in treating severe osteoarthritis, nearly 25% of patients were dissatisfied after TKA, and up to 30% had anterior knee pain or painless noise [1]. MR prostheses were designed based on the J curve theory and have been the most widely utilized prostheses since the 1980s [6, 7]. SR prostheses were designed based on the theory that the actual FEA of the posterior femur condyles was considered a single-radius axis of a cylinder for flexion and extension of the knee through 10–120°. SR prostheses provide a more posterior flexion axis and an increased extensor moment arm to relieve the tension of the quadriceps. Additionally, SR designs maintain middle range stabilization and reduce the paradoxical anterior femoral movement to alleviate AKP.

Our study reported that the SR prostheses gave less anterior knee pain than did the MR prostheses. This is in line with Hamilton et al. [11], who compared 75 SR (Triathlon) with 90 MR prostheses, reporting that the SR designs reduced the worst daily pain (*P* = 0.003) over 3 years of follow-up; however, Oxford Knee Scores (*P* = 0.09) and timed functional performance tasks (*P* = 0.23) did not reach statistical significance. Similarly, Palmer et al. [23] reported that 66.3% of patients with the SR prostheses experienced no pain, less than 54.4% with the MR prostheses. Anterior knee pain had related factors in some of the implant designs. Overstuffing of the knee joint, patella impingement, and instability of the knee may result to pain [24]. Similar sizes of the femoral implants were utilized in both groups, and patellar resurfacing was not utilized in neither patient in our study. Theoretically, SR provided a lateral flexion axis so the crepitus between patella and femoral implants may be less than MR. SR increased extensor moment arm to relieve the tension of the quadriceps as to led to better quadriceps efficiency. In the MR femoral implants, the length of the ligament changes at mid-flexion as the momentary axis changes from a long one to a short one, which may cause in instability, while the SR femoral design has a fixed main axis which maintains the tension of the collateral ligaments during the movement. All of these may explain our follow-up discovery.

SR had theoretical advantages that were likely to translate into satisfactory improved clinical outcomes. We did not observe significant improvement in terms of other clinical, radiological, and survival results when comparing the SR with the MR prostheses in TKA. Several possible reasons could explain this.

First, the clinical scales traditionally used to generate orthopedic results such as HSS and KSS score may not be sensitive enough to illuminate differences in prostheses designs [25]. Mahoney et al. [10] compared 83 patients with SR (Scorpio) to 101 patients with MR prostheses and reported that significantly more patients in the SR group were able to rise from a 16-in. chair without using their arms starting at 6 weeks and the difference was maintained through 2 years; however, there were no differences in the KSS scores between the two groups at 2 years. Jo et al. [14] compared 50 patients with SR (Scorpio NRG) and 50 patients with MR, using a navigation system to measure stability during 0°, 30°, 60°, and 90° of flexion, and reported that the SR group had better intraoperative stability, especially improved midrange stability from 60 to 90°, but no significant differences in the HSS scores or WOMAC scores. Larsen et al. [16] compared 16 patients with SR (Scorpio) to 16 patients with MR prostheses, using computer navigation for gait analysis, and reported that the SR implant had better kinematic properties at 1-year follow-up than did the MR prostheses. MR prostheses remained more extended and had decreased power absorption during weight acceptance than did the SR prostheses. However, both surgical groups had similar KSS for knee and functional scores at the 1-year follow-up. Therefore, any advantages of the SR prostheses in terms of muscular recovery, component stability, or gait cycle may not be accurately reflected in the conventional function scales.

Second, soft tissue and gap balance are influenced by many factors, including different types of osteotomies and the effect of releasing the tight medial-lateral structures. Therefore, the stability of the knee could not be completely guaranteed by the SR prosthesis. Stoddard et al. [15] compared eight patients with SR (Triathlon) to eight patients with MR prostheses using a computer navigation system to measure the stability and reported that significant differences were not found between the types of femur design and that mid-range instability may have been related to unrecognized ligament laxity. Therefore, considering the complexity and multi-step process of the soft tissue balance, SR prostheses did not guarantee the stability of the knee at all angles.

Our study had several limitations. First, it was a retrospective cohort study such that selection bias would be expected. Nevertheless, our study reported long-term follow-up with a large population of consecutive patients. Second, more accurate tests are required to increase the sensitivity of the clinical and functional results.

## Conclusion

Both SR and MR posterior-stabilized prostheses can lead to satisfactory outcomes. The SR prosthesis design gave less anterior knee pain than did the MR prostheses. The two prostheses showed no differences in terms of clinical scales, radiological results, satisfaction rate, or survival results at long-term follow-up, requiring more accurate measurements.

## Abbreviations

AKP: Anterior knee pain
CR: Cruciate-retaining
FEFA: Flexion/extension femoral angle
HSS: Hospital for Special Surgery
KA: Kinematic alignment
LDFA: Lateral distal femoral angle
MA: Mechanical alignment
MCL: Medial collateral ligament
MDTA: Medial proximal tibia angle
MR: Multi-radius
PCL: Posterior cruciate ligaments
ROM: Range of motion
SF-12 Scale: Short Form-12 health survey
SR: Single-radius
TEA: Trans-epicondylar axis
TKA: Total knee arthroplasty
TSA: Tibial slope angle
VAS: Visual analog scale
